# Preexisting and de novo humoral immunity to SARS-CoV-2 in humans

**DOI:** 10.1126/science.abe1107

**Published:** 2020-11-06

**Authors:** Kevin W. Ng, Nikhil Faulkner, Georgina H. Cornish, Annachiara Rosa, Ruth Harvey, Saira Hussain, Rachel Ulferts, Christopher Earl, Antoni G. Wrobel, Donald J. Benton, Chloe Roustan, William Bolland, Rachael Thompson, Ana Agua-Doce, Philip Hobson, Judith Heaney, Hannah Rickman, Stavroula Paraskevopoulou, Catherine F. Houlihan, Kirsty Thomson, Emilie Sanchez, Gee Yen Shin, Moira J. Spyer, Dhira Joshi, Nicola O’Reilly, Philip A. Walker, Svend Kjaer, Andrew Riddell, Catherine Moore, Bethany R. Jebson, Meredyth Wilkinson, Lucy R. Marshall, Elizabeth C. Rosser, Anna Radziszewska, Hannah Peckham, Coziana Ciurtin, Lucy R. Wedderburn, Rupert Beale, Charles Swanton, Sonia Gandhi, Brigitta Stockinger, John McCauley, Steve J. Gamblin, Laura E. McCoy, Peter Cherepanov, Eleni Nastouli, George Kassiotis

**Affiliations:** 1 Retroviral Immunology, The Francis Crick Institute, London NW1 1AT, UK.; 2 Chromatin Structure and Mobile DNA Laboratory, The Francis Crick Institute, London NW1 1AT, UK.; 3 Worldwide Influenza Centre, The Francis Crick Institute, London NW1 1AT, UK.; 4 Signalling and Structural Biology Laboratory, The Francis Crick Institute, London NW1 1AT, UK.; 5 Structural Biology of Disease Processes Laboratory, The Francis Crick Institute, London NW1 1AT, UK.; 6 Structural Biology STP, The Francis Crick Institute, London NW1 1AT, UK.; 7 Flow Cytometry STP, The Francis Crick Institute, London NW1 1AT, UK.; 8 Peptide Chemistry, The Francis Crick Institute, London NW1 1AT, UK.; 9 Cell Biology of Infection Laboratory, The Francis Crick Institute, London NW1 1AT, UK.; 10 Cancer Evolution and Genome Instability Laboratory, The Francis Crick Institute, London NW1 1AT, UK.; 11 Neurodegeneration Biology Laboratory, The Francis Crick Institute, London NW1 1AT, UK.; 12 AhRimmunity Laboratory, The Francis Crick Institute, London NW1 1AT, UK.; 13 University College London Hospitals (UCLH) NHS Trust, London NW1 2BU, UK.; 14 Division of Infection and Immunity, University College London (UCL), London WC1E 6BT, UK.; 15 Department of Population, Policy and Practice, Great Ormond Street Institute for Child Health (ICH), UCL, London WC1N 1EH, UK.; 16 Public Health Wales, University Hospital of Wales, Cardiff CF14 4XW, UK.; 17 Centre for Adolescent Rheumatology Versus Arthritis at UCL, UCLH, Great Ormond Street Hospital (GOSH), London WC1N 3JH, UK.; 18 Centre for Rheumatology Research, Division of Medicine, UCL, London, WC1E 6BT, UK.; 19 UCL Great Ormond Street Institute for Child Health (ICH), UCL, London WC1N 1EH, UK.; 20 Department of Medicine, Faculty of Medicine, Imperial College London, London W2 1PG, UK.

## Abstract

Zoonotic introduction of novel coronaviruses may encounter preexisting immunity in humans. Using diverse assays for antibodies recognizing SARS-CoV-2 proteins, we detected preexisting humoral immunity. SARS-CoV-2 spike glycoprotein (S)–reactive antibodies were detectable using a flow cytometry–based method in SARS-CoV-2–uninfected individuals and were particularly prevalent in children and adolescents. They were predominantly of the immunoglobulin G (IgG) class and targeted the S2 subunit. By contrast, SARS-CoV-2 infection induced higher titers of SARS-CoV-2 S–reactive IgG antibodies targeting both the S1 and S2 subunits, and concomitant IgM and IgA antibodies, lasting throughout the observation period. SARS-CoV-2–uninfected donor sera exhibited specific neutralizing activity against SARS-CoV-2 and SARS-CoV-2 S pseudotypes. Distinguishing preexisting and de novo immunity will be critical for our understanding of susceptibility to and the natural course of SARS-CoV-2 infection.

## Antibodies predating infection

Immunological memory after infection with seasonal human coronaviruses (hCoVs) may potentially contribute to cross-protection against severe acute respiratory syndrome coronavirus 2 (SARS-CoV-2). Ng *et al.* report that in a cohort of 350 SARS-CoV-2–uninfected individuals, a small proportion had circulating immunoglobulin G (IgG) antibodies that could cross-react with the S2 subunit of the SARS-CoV-2 spike protein (see the Perspective by Guthmiller and Wilson). By contrast, COVID-19 patients generated IgA, IgG, and IgM antibodies that recognized both the S1 and S2 subunits. The anti-S2 antibodies from SARS-CoV-2–uninfected patients showed specific neutralizing activity against both SARS-CoV-2 and SARS-CoV-2 S pseudotypes. A much higher percentage of SARS-CoV-2–uninfected children and adolescents were positive for these antibodies compared with adults. This pattern may be due to the fact that children and adolescents generally have higher hCoV infection rates and a more diverse antibody repertoire, which may explain the age distribution of COVID-19 susceptibility.

*Science*, this issue p. 1339; see also p. 1272

## Teaser

SARS-CoV-2 neutralizing antibodies can be found in some uninfected individuals—predominantly children and adolescents.

Immune cross-reactivity among seasonally spreading human coronaviruses (HCoVs) has long been hypothesized to provide effective but transient cross-protection against distinct HCoVs (*1*, *2*). To determine the degree of cross-reactivity between HCoVs and SARS-CoV-2, we developed a flow cytometry–based assay for SARS-CoV-2–binding antibodies. The main target for such antibodies is the spike glycoprotein (S), which is proteolytically processed into the S1 and S2 subunits, mediating target cell attachment and entry, respectively.

**Fig. 1:**
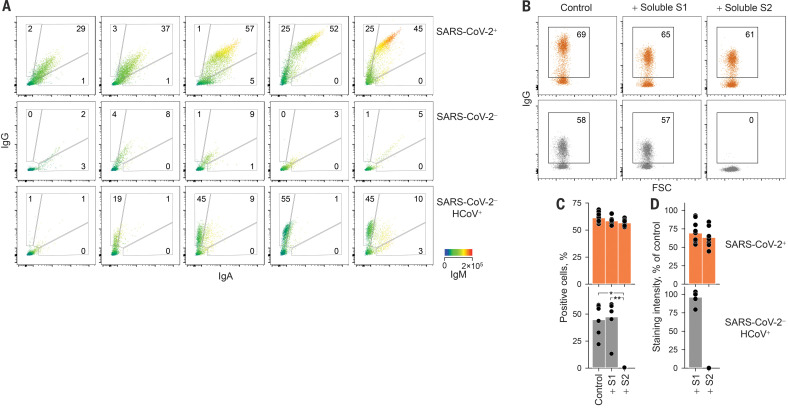
Flow cytometric detection and specificity of antibodies reactive with SARS-CoV-2 S. (**A**) Detection of IgG, IgA, and IgM in five individuals from each indicated group. IgM levels are indicated by a heatmap. (**B** to **D**) Inhibition of SARS-CoV-2 S binding of sera from SARS-CoV-2–infected (SARS-CoV-2^+^, *n* = 10) or SARS-CoV-2–uninfected (SARS-CoV-2^−^ HCoV^+^, *n* = 6) patients by soluble S1 or S2. (B) Flow cytometry profile of one representative patient per group. (C) Mean frequency of positive cells. *P = 0.015; **P = 0.006, one-way analysis of variance (ANOVA) on ranks. (D) Mean staining intensity [mean fluorescence intensity (MFI) of sample as a percentage of negative control MFI]. In (C) and (D), dots represent individual samples from one of three similar experiments.

The S1-specific CR3022 antibody stained a smaller percentage of SARS-CoV-2 S–expressing human embryonic kidney (HEK) 293T cells and with lower intensity than COVID-19 convalescent sera (fig. S1), indicating that polyclonal immunoglobulin G (IgG) antibodies targeted a wider range of epitopes naturally processed and displayed on these cells. This assay also detected SARS-CoV-2 S–reactive IgM and IgA antibodies in COVID-19 convalescent sera (fig. S2). Indeed, the presence of SARS-CoV-2 S–reactive antibodies of all three Ig classes (IgG^+^IgM^+^IgA^+^) distinguished COVID-19 sera from control sera with a high degree of sensitivity and specificity (Fig. 1A and fig. S3). All 156 seroconverted COVID-19 patients had contemporaneous IgG, IgM, and IgA responses to SARS-CoV-2 S throughout the observation period, with the exception of two patients who only had IgG antibodies (figs. S4 and S5). One of these patients was a bone marrow transplantation recipient who experienced HCoV infection 1 month before SARS-CoV-2 infection (fig. S6). Unexpectedly, a small proportion of SARS-CoV-2–uninfected patients sampled before or during the early spread of SARS-CoV-2 in the United Kingdom (table S1) also had SARS-CoV-2 S–binding IgG antibodies, but not IgM or IgA antibodies (Fig. 1A), suggesting the presence of cross-reactive immunological memory.

The S2 subunit exhibits a higher degree of homology among coronaviruses than S1 (fig. S7) and was likely the main target of cross-reactive antibodies. Competition with recombinant soluble S1 or S2 at doses that blocked binding of specific monoclonal antibodies (fig. S8) did not affect the frequency of cells stained with COVID-19 patient sera, although the intensity of staining was reduced by 31 and 37%, respectively (Fig. 1, B to D), indicating recognition of both S1 and S2. By contrast, soluble S2 completely abolished staining with SARS-CoV-2–uninfected patient sera, whereas soluble S1 had no effect (Fig. 1, B to D). Thus, SARS-CoV-2–uninfected patient sera cross-react with SARS-CoV-2 S2, and COVID-19 patient sera additionally recognize S1.

**Fig. 2:**
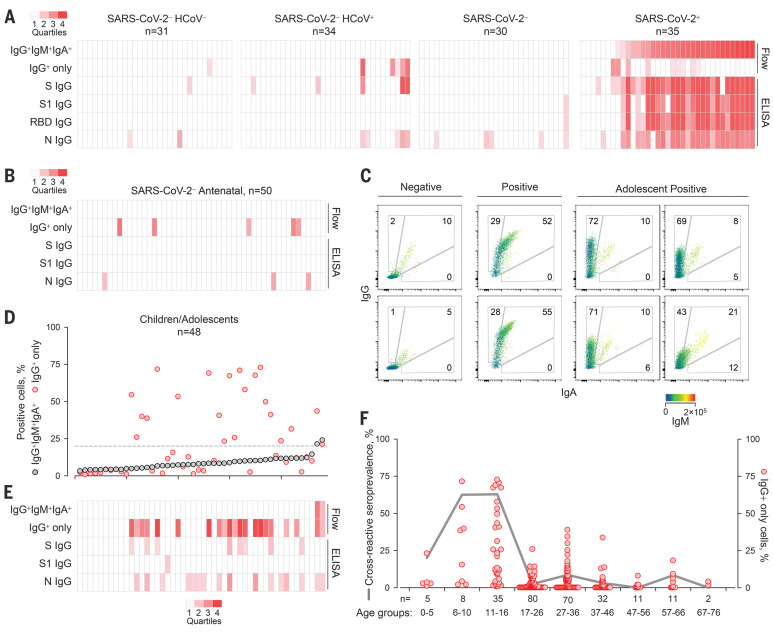
Prevalence of SARS-CoV-2 S–cross-reactive antibodies detected by different methods. (**A**) Flow cytometry and ELISA results for each sample in cohorts A and C to E listed in table S1. (**B**) Flow cytometry and ELISA results for serum samples from SARS-CoV-2–uninfected pregnant women. (**C** to **E**) SARS-CoV-2 S–cross-reactive antibodies in healthy children and adolescents. (C) Representative flow cytometry profiles of seronegative donors (Negative) or COVID-19 patients (Positive) and of SARS-CoV-2–uninfected adolescents with SARS-CoV-2 cross-reactive antibodies. (D) Frequency of cells stained with all three antibody classes (IgG^+^IgM^+^IgA^+^) or only with IgG (IgG^+^) ranked by their IgG^+^IgM^+^IgA^+^ frequency. The dashed line denotes the assay sensitivity cutoff. (E) Flow cytometry and ELISA results for each sample. (**F**) Prevalence of SARS-CoV-2 S–cross-reactive antibodies in the indicated age groups (line) and frequency of cells that stained only with IgG (dots) in all samples for which the date of birth was known. The heatmaps in (A), (B), and (E) represent the quartile values above each assay’s technical cutoff.

SARS-CoV-2 S–reactive IgG antibodies were detected by flow cytometry in five of 34 SARS-CoV-2–uninfected individuals with HCoV infection confirmed by reverse transcription–quantitative polymerase chain reaction, as well as in one of 31 individuals without recent HCoV infection (Fig. 2A and fig. S4A). This suggested that cross-reactivity may have persisted from earlier HCoV infections rather than having been induced by the most recent one.

To confirm antibody cross-reactivity using an independent assay, we developed enzyme-linked immunosorbent assays (ELISAs) using recombinant SARS-CoV-2–stabilized trimeric S ectodomain, S1, receptor-binding domain (RBD), or nucleoprotein (N). Rates of IgG seropositivity by SARS-CoV-2 S1–coated ELISA were congruent with, but generally lower than, those by flow cytometry (fig. S9). The three SARS-CoV-2–uninfected individuals with the highest cross-recognition of S by flow cytometry, plus an additional four individuals, had ELISA-detectable IgG antibodies against the SARS-CoV-2 S ectodomain, as well as N (Fig. 2A and fig. S4, B to D). By contrast, none of the control samples had ELISA-detectable IgG antibodies against the less-conserved SARS-CoV-2 S1 or RBD (Fig. 2A and fig. S4, B to D).

The prevalence of such cross-reactive antibodies was further examined in additional healthy donor cohorts (table S1). Among 50 SARS-CoV-2–uninfected pregnant women sampled in May of 2018, five showed evidence for SARS-CoV-2 S–reactive IgG antibodies, but not IgM or IgA antibodies (Fig. 2B and fig. S10). In a separate cohort of 101 SARS-CoV-2–uninfected donors sampled in May of 2019, three had SARS-CoV-2 S–reactive IgG antibodies (fig. S11) that did not correlate with antibodies to the diverse viruses and bacteria also present in several of these samples. SARS-CoV-2 S–reactive IgM and IgA were also detected in two of these donors, albeit at considerably lower levels than in COVID-19 patients (fig. S11), suggestive of recent or ongoing response. In an additional cohort of 13 donors recently infected with HCoVs, only one had SARS-CoV-2 S–reactive IgG antibodies, and these were at very low levels (fig. S12). This suggested that their emergence was not simply a common transient event after each HCoV infection in this age group (median age 51 years; table S1). Instead, given that HCoV-reactive antibodies are present in virtually all adults (*3*–*5*), the rarity of SARS-CoV-2 S cross-reactivity (16 of 302; 5.29%) indicates additional requirements such as random B cell receptor repertoire focusing or frequency of HCoV infection rather than time since the last HCoV infection. Indeed, the frequency of HCoV infection displays a characteristic age distribution, being the highest in children and adolescents (*1*, *4*–*8*). We therefore examined a cohort of younger SARS-CoV-2–uninfected healthy donors (age 1 to 16 years; table S1) sampled between 2011 and 2018. At least 21 of these 48 donors had detectable levels of SARS-CoV-2 S–reactive IgG antibodies (Fig. 2, C to E), whereas only one of an additional cohort of 43 young adults (age 17 to 25 years; table S1) had such antibodies (Fig. 2F). Staining with sera from SARS-CoV-2–uninfected children and adolescents was specific to HEK293T cells expressing SARS-CoV-2 S, but not the unrelated HERV-K113 envelope glycoprotein, and was outcompeted by soluble SARS-CoV-2 S2 (fig. S13). The prevalence of SARS-CoV-2 S–reactive IgG antibodies peaked at 62% between 6 and 16 years of age (Fig. 2F), when HCoV seroconversion in this age group also peaks (*3*, *4*, *6*, *7*), and was significantly higher than in adults (*P* < 0.00001, Fisher’s exact test).

**Fig. 3:**
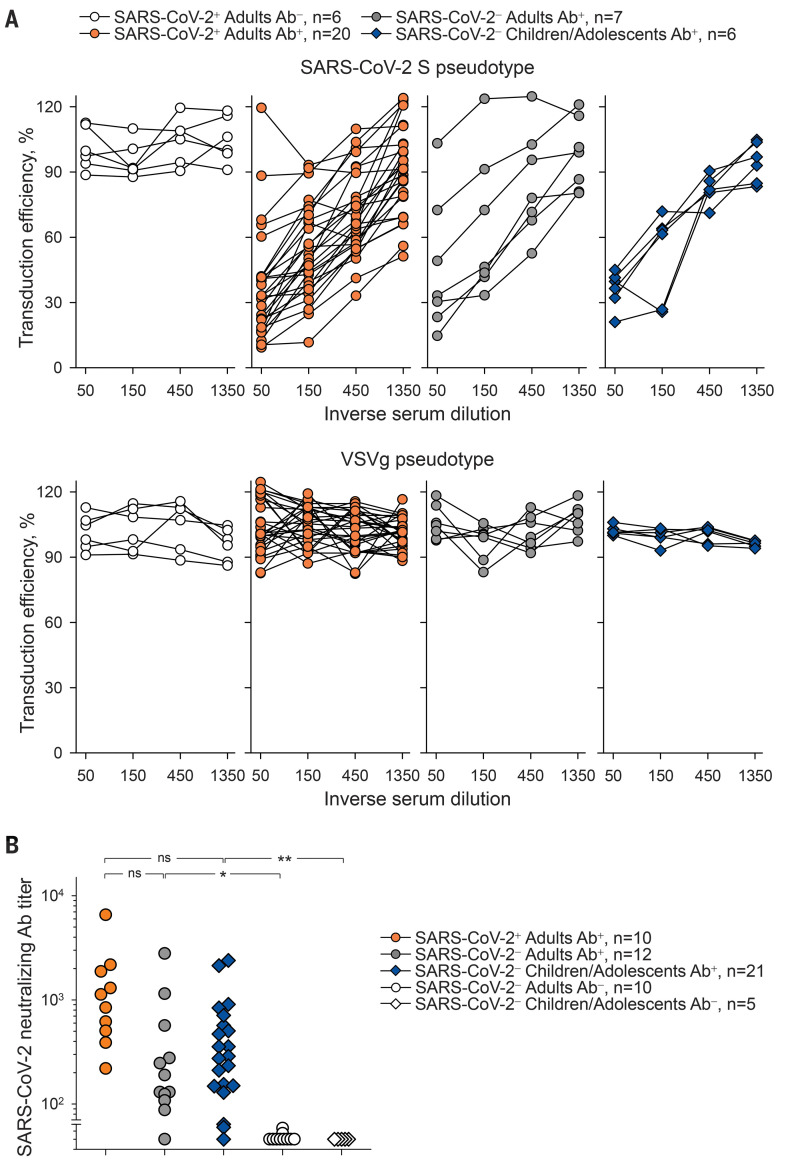
Neutralization of SARS-CoV-2 S pseudotypes and authentic SARS-CoV-2 by SARS-CoV-2–infected and –uninfected patient sera. (**A**) Inhibition of transduction efficiency of SARS-CoV-2 S or VSVg pseudotypes by adult COVID-19 patients who seroconverted (SARS-CoV-2^+^ Adults Ab^+^) or not (SARS-CoV-2^+^ Adults Ab^−^) and SARS-CoV-2–uninfected adult donors (SARS-CoV-2^−^ Adults Ab^+^) or children and adolescent donors (SARS-CoV-2^−^ Children/Adolescents Ab^+^) with SARS-CoV-2 S–binding antibodies. Each line is an individual serum sample. (**B**) Authentic SARS-CoV-2 neutralization titers of sera from the same donors as in (A), as well as SARS-CoV-2–uninfected donors without SARS-CoV-2 S–binding antibodies (Ab^−^). Dots represent individual samples. *P = 0.037; **P = 0.014; ns, not significant by one-way ANOVA on ranks.

To determine the potential consequences of antibody cross-reactivity, we examined the ability of preexisting antibodies to inhibit SARS-CoV-2 entry into HEK293T cells (fig. S14 and supplementary text). Although not expected to directly inhibit RBD-mediated cell attachment, S2-targeting antibodies that can neutralize SARS-CoV-2 have recently been discovered (*9*, *10*). HEK293T cell infection with SARS-CoV-2 S pseudotypes was efficiently inhibited by sera from seroconverted (Ab^+^) COVID-19 patients, but not from those who had not yet seroconverted (Ab^−^) (Fig. 3A). Sera from SARS-CoV-2–uninfected donors with SARS-CoV-2 S–reactive antibodies also neutralized these pseudotypes, whereas none of the sera neutralized vesicular stomatitis virus (VSV) glycoprotein pseudotypes (Fig. 3A). Comparable neutralization of SARS-CoV-2 S pseudotypes was also observed with sera from SARS-CoV-2–uninfected adolescents (Fig. 3A). Moreover, most of the sera from SARS-CoV-2–uninfected donors with flow cytometry–detectable cross-reactive antibodies also neutralized authentic SARS-CoV-2 infection of Vero E6 cells, albeit on average less potently than COVID-19 patient sera (Fig. 3B). By contrast, sera from SARS-CoV-2–uninfected patients without cross-reactive antibodies exhibited no neutralizing activity (Fig. 3B). Antiviral antibodies may also enhance viral entry by Fc receptor–mediated antibody-dependent enhancement. However, entry of SARS-CoV-2 S pseudotypes was not enhanced by either COVID-19 patient sera or SARS-CoV-2–uninfected patient sera in FcγRIIA-expressing K-562 cells (fig. S15).

**Fig. 4:**
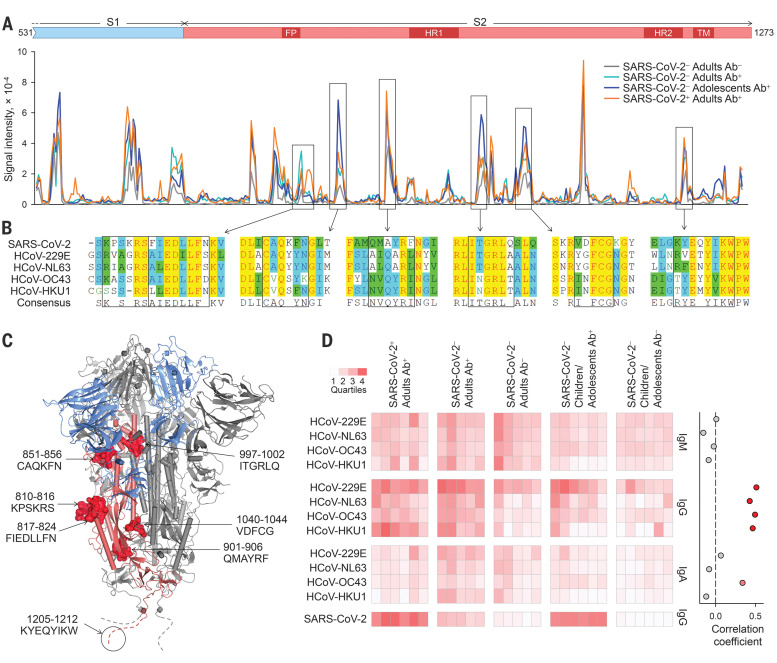
Mapping of cross-reactive epitopes in SARS-CoV-2 S. (**A**) Signal intensity for each overlapping peptide along the length of SARS-CoV-2 S covered in the peptide arrays using pooled sera with (Ab^+^) or without (Ab^−^) flow cytometry–detectable SARS-CoV-2 S–reactive antibodies. Differentially recognized peaks are boxed. (**B**) Alignment of the amino acid sequences of SARS-CoV-2 and HCoV S glycoproteins. Boxes indicate predicted core epitopes. (**C**) Mapping of predicted epitopes targeted on the trimeric SARS-CoV-2 spike. The S1 (blue) and S2 (pink) subunits of one monomer are colored. Epitopes are shown for one monomer; the circled dashed line represent the membrane proximal region not present in the structure. (**D**) Left: Reactivity with the S glycoproteins of each HCoV of the indicated sera with (Ab^+^) or without (Ab^−^) flow cytometry–detectable SARS-CoV-2 S–reactive antibodies as determined by flow cytometry. Each column is an individual sample. Rows depict the staining for each antibody class. Right: Correlation coefficients between percentages of IgG staining for SARS-CoV-2 S and IgG, IgM, and IgA staining for each HCoV S glycoprotein.

Collectively, these findings highlight functionally relevant antigenic epitopes conserved within the S2 subunit. Over its entire length, SARS-CoV-2 S exhibits marginally closer homology with the S proteins of the betacoronaviruses HCoV-OC43 and HCoV-HKU1 than with the alphacoronaviruses HCoV-NL63 and HCoV-229E (fig. S16A). To probe shared epitopes, we constructed overlapping peptide arrays spanning the last 743 amino acids of SARS-CoV-2 S (fig. S16B). Multiple putative epitopes were differentially recognized by sera with cross-reactive antibodies (Ab^+^), were reasonably conserved, and most mapped to the surface of S2 (Fig. 4, A and B, and table S2). An epitope overlapping the S2 fusion peptide was also recently identified as being cross-reactive with the corresponding peptides from HCoV-OC43 and HCoV-229E (*11*). Cross-reactivity with the identified epitopes was further supported by ELISAs coated with synthetic peptides (fig. S17).

As expected (*3*–*5*), reactivity with one or more HCoVs was detectable by flow cytometry in all sera (Fig. 4D and fig. S18). However, IgG and IgA reactivity against HCoVs was higher in SARS-CoV-2–uninfected adults with SARS-CoV-2–reactive IgG compared with those without (*P* = 1.4 × 10^–6^ for IgG and *P* = 0.017 for IgA, Student’s *t* test) and in SARS-CoV-2–uninfected children or adolescents with SARS-CoV-2–reactive IgG compared with those without (*P* = 0.010 for IgG and *P* = 0.021 for IgA, Student’s *t* test) (Fig. 4D), supporting a direct link between the two. Accordingly, IgG reactivity against each HCoV type was independently correlated with the presence of SARS-CoV-2–reactive antibodies (Fig. 4D).

Our results from multiple independent assays demonstrate the presence of preexisting antibodies recognizing SARS-CoV-2 in uninfected individuals. Identification of conserved epitopes in S2 targeted by neutralizing antibodies may hold promise for a universal vaccine protecting against current as well as future CoVs. Together with preexisting T cell (*12*–*14*) and B cell (*10*, *15*) memory, antibody cross-reactivity between seasonal HCoVs and SARS-CoV-2 may have important ramifications for natural infection. Epidemiological studies of HCoV transmission suggest that cross-protective immunity is unlikely to be sterilizing or long-lasting (*8*), which is also supported by repeated reinfection (*2*, *16*). Nevertheless, prior immunity induced by one HCoV can reduce the transmission of homologous and heterologous HCoVs and ameliorate the symptoms when transmission is not prevented (*1*, *2*). A possible modification of COVID-19 severity by prior HCoV infection may account for the age distribution of COVID-19 susceptibility, in which higher HCoV infection rates in children than in adults (*4*, *6*) correlate with relative protection from COVID-19 (*17*) and may also shape seasonal and geographical patterns of transmission. It is imperative that any effect, positive or negative, of preexisting HCoV-elicited immunity on the natural course of SARS-CoV-2 infection be fully delineated.

## Supplementary Materials

science.sciencemag.org/content/370/6522/1339/suppl/DC1 Materials and Methods Supplementary Text Figs. S1 to S18 Tables S1 and S2 References (*18*–*40*) MDAR Reproducibility Checklist

## Appendix

View/request a protocol for this paper from *Bio-protocol*.
